# The Risk of Atrial Fibrillation Increases with Earlier Onset of Obesity: A Mendelian Randomization Study

**DOI:** 10.7150/ijms.72334

**Published:** 2022-08-08

**Authors:** Yingchao Zhou, Lingfeng Zha, Silin Pan

**Affiliations:** 1 Heart Center, Women and Children's Hospital, Qingdao University, Qingdao, China.; 2 Department of Cardiology, Union Hospital, Tongji Medical College, Huazhong University of Science and Technology, Wuhan, China.

**Keywords:** Obesity, Atrial fibrillation, Mendelian randomization, Genome-wide association study, Genetics

## Abstract

**Background:** Obesity is a well-established risk factor for atrial fibrillation (AF). Previous epidemiological research on obesity and AF often focused on adult populations and now broadened to earlier in life. Therefore, this study aimed to determine the relationships between obesity at different periods of life and the risk of AF.

**Methods:** A two-sample Mendelian randomization (MR) study design using summarised data from 6 genome-wide association studies (GWASs) was employed in this study. Single nucleotide polymorphisms (SNPs) associated with adult obesity, childhood obesity, childhood body mass index (BMI), waist-to-hip ratio adjusted for BMI (WHRadjBMI), birth weight and AF were independently retrieved from large-scale GWASs. For SNP identification, the genome-wide significance threshold was set at *p* <5.00×10^-8^. To obtain causal estimates, MR analysis was conducted using the inverse variance-weighted (IVW) method. The weighted median, MR-Egger methods and MR-robust adjusted profile score (MR-RAPS) were used to evaluate the robustness of MR analysis.

**Results:** A total of 204 SNPs were identified as the genetic instrumental variables (5 SNPs for childhood obesity, 13 SNPs for childhood BMI, 137 SNPs for birth weight, 35 SNPs for adult WHRadjBMI, and 14 SNPs for adult obesity). The results of MR analysis demonstrated that the genetically predicted adult obesity, childhood BMI, and birth weight were associated with AF risk. Notably, a 1 unit standard deviation (1-SD) increase in adult obesity was related to a 13% increased risk of AF [*p*=6.51×10^-7^, OR, 1.13 (95% CI, 1.08-1.19)], a 1-SD increase in childhood BMI was related to a 18% increased risk of AF [*p*=1.77×10^-4^, OR, 1.18 (95% CI, 1.08-1.29)], and a 1-SD increase in birth weight was related to a 26% increased risk of AF [*p*=1.27×10^-7^, OR, 1.26 (95% CI, 1.16-1.37)]. There was no evidence of pleiotropy or heterogeneity between the MR estimates obtained from multiple SNPs.

**Conclusion:** Our study reveals the association of genetic susceptibility to obesity with a higher risk of AF. Moreover, an earlier age at obesity was associated with an increased risk of AF. Therefore, public awareness of the dangers of obesity and active early weight control may prevent the development of AF.

## Introduction

Atrial fibrillation (AF) is one of the most common arrhythmias, with high mortality and morbidity rates 1. The prevalence of AF is expected to be 1-2% 2, which will increase significantly in the next 30-50 years due to population aging 3, and is likely to become a greater public health burden 4. Known risk factors increase the incidence of AF include non-modifiable risk factors (e.g., advancing age, racial/ethnic differences and genetic predisposition) 5, modifiable risk factors (e.g., body mass index [BMI], height, hypertension, diabetes mellitus, obstructive sleep apnea, myocardial infarction, heart failure, and smoking) 6 and modern lifestyle. Modifying the risk factors for AF is important when trying to decrease risk of developing AF.

The increasing number of people with obesity has resulted in an increasing prevalence of AF. It is important to recognize that obesity as an independent risk factor for AF in the Mendelian randomization (MR) 7 and observational study designs 8. In fact, obesity increased the risk of AF by 50% 9 and every 1-unit increase in BMI increased AF risk by 4-5% 10. There is a well-established epidemiological association between obesity and AF. However, research using measurements of BMI at a single point in time fails to assess the cumulative effect of obesity over the life course on AF development 11. Obesity more likely established early in life endure until adulthood 12. Consistent with these finding, Hindricks G et al. found that overweight newborns (> 4 kg) may have a higher risk of AF in adulthood than normal-weight newborns 13. Therefore, early detection of these changes in individuals is helpful for the optimal management of AF in clinical practice.

In this study, we simultaneously investigated the association between different stages of obesity (e.g. adult obesity, childhood obesity, birth weight) and AF risk using a two-sample MR design, In the MR analysis, single nucleotide polymorphisms (SNPs) are often used as proxies for the exposure of interest 14, 15. The measurement of SNPs is easy and they remain unchanged during lifetime, Moreover, the prediction accuracy of genetic factors is especially high among young children 16. Therefore, the MR approach is less likely to be affected by unmeasured confounding bias and reverse causation. The study highlights the importance of obesity prevention and treatment at younger ages to tackle the AF epidemic.

## Methods

### Research concept

**Figure 1:**
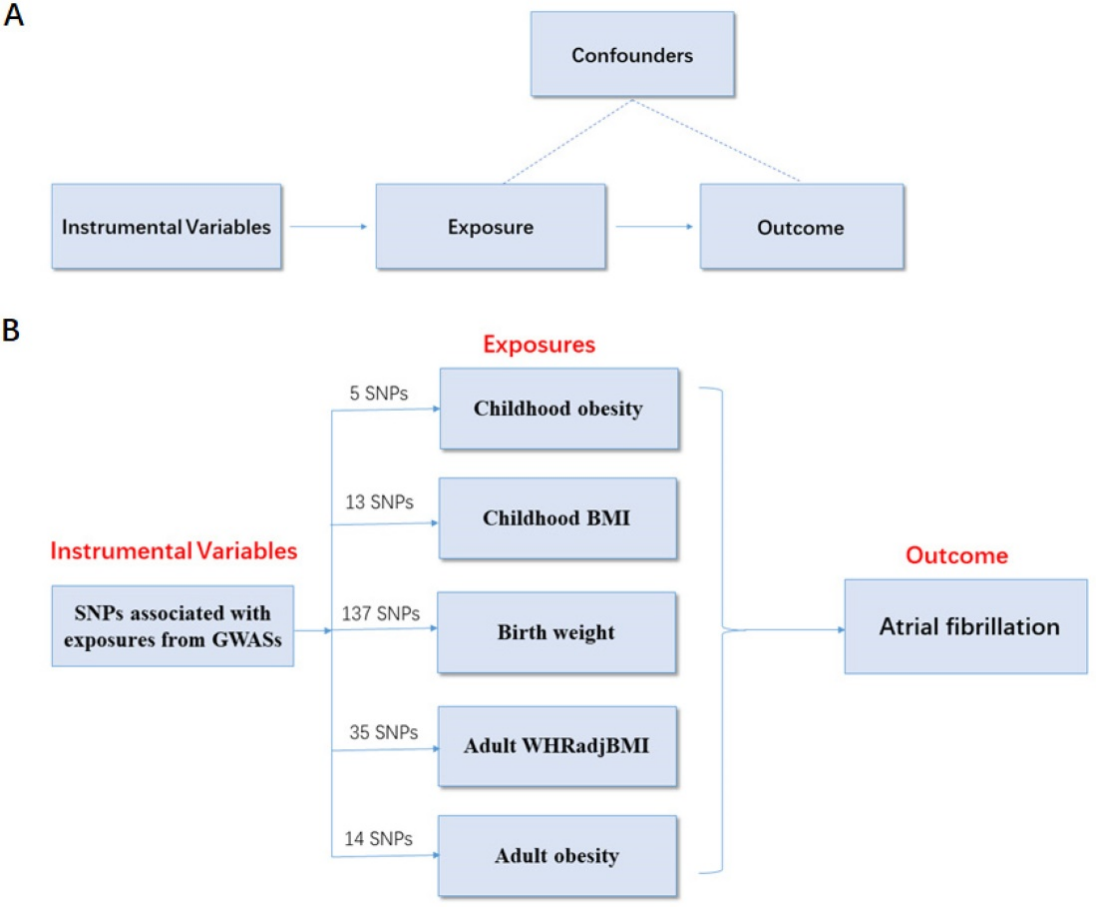
** MR study design principles and design scheme.** (**A**) Design principles; (**B**) design scheme. The two-sample MR analysis assumes that the instrument associates with the exposure; the instrument does not influence the outcome through some pathway other than the exposure; and the instrument does not associate with confounders. SNP, single-nucleotide polymorphism; BMI, body mass index; WHRadjBMI, waist-to-hip ratio adjusted for body mass index; MR, Mendelian randomization.

To determine the causal relationship between obesity-related traits and AF risk, a two-sample MR analysis was conducted using summarized data from 6 genome-wide association studies (GWASs) based on the following assumptions. (i) Assumptions for relevance: genetic instrumental variables are closely related to obesity. (ii) Assumptions for independence: the variables are independent of confounders that can influence the association between obesity and AF risk. (iii) Assumptions for exclusion restriction: the variables are associated with the risk of AF only through obesity (Figure 1A).

### Data sources

#### Exposure: obesity-related traits

Genetically predicted obesity-related traits were used as an exposure in this study. The adult obesity-associated SNPs were retrieved from the largest GWAS meta-analysis (*n* = 263,407, European population, adult obesity was defined as BMI ≥ 30 kg/m^2^, age >19 years) 17. The SNPs related to adult waist-to-hip ratio adjusted for BMI (WHRadjBMI) were extracted from the GWAS meta-analysis (*n* = 224,459, European population, age >19 years). The childhood obesity-associated SNPs were retrieved from the childhood obesity GWAS meta-analysis (*n* = 13,848, European population, the childhood normal weight controls and childhood obesity cases were defined as BMI < 50^th^ and ≥ 95^th^ percentiles, respectively, age of the cases ranged from 2 to 10 years) 18. The SNPs related to childhood BMI were acquired from the childhood BMI GWAS meta-analysis (*n* = 47,541, European population, age from 2 to 18 years) 19. The SNPs related to birth weight were obtained from the birth weight GWAS meta-analysis (*n* = 321,223, 92.81% European population) 20. The information of each GWAS is shown in Table 1.

#### Outcome: AF

The primary outcome of this study was AF. Data on the association between SNPs and AF were obtained from the most recent GWAS of AF 21, which included 522,744 controls and 65,446 cases from four resources, including 18,000 overlapping cases from another AF GWAS by Nielsen et al. 22. Most of the subjects (91.37%) were of European descent (Table 1). They also carried out a meta-analysis in the non-overlapping individuals from these two large studies with a resulting total of more than 93,000 AF cases and up to 1 million controls making their study as the hitherto largest GWAS meta-analysis for AF. AF was diagnosed based on the International Classification of Diseases (ICD)-9/10. Cases included participants with paroxysmal or permanent AF, or atrial flutter. The age for the case of AF is > 19 years, with the mean (SD) age is 45±26 years.

### SNP data analysis

To ensure a strong association between the genetic instrumental variables and obesity-related traits, the abovementioned GWAS datasets were used to identify SNPs with genome-wide significant (*p* < 5.00×10^-8^, r^2^ < 0.80). Each SNP associated with obesity-related traits was assessed for its association with AF, and SNPs with pleiotropic associations were excluded. To ensure consistency, we adjusted the effect direction of SNPs in all GWAS. As a result, there were 5 SNPs related to childhood obesity, 13 SNPs related to childhood BMI, 137 SNPs related to birth weight, 35 SNPs related to adult WHRadjBMI, and 14 SNPs associated with adult obesity. These SNPs were included in the subsequent MR analysis of AF risk (Figure 1B).

### MR analysis

In this study, the causal relationship between obesity-related SNPs and AF risk was determined using the inverse variance-weighted (IVW) method, which was considered as the most reliable indicator. In the follow-up sensitivity analyses, weighted median, MR-Egger methods and the MR-robust adjusted profile score (MR-RAPS) 23 were used to evaluate the robustness of MR analysis. Horizontal pleiotropy was identified when the SNPs affect the outcome through pathways other than the exposure, which violation of MR. Methods such as MR-Egger and weighted median methods could explore and account for the impact of horizontal pleiotropy. The Egger regression intercept is an estimate of the magnitude of horizontal pleiotropy. If there was no evidence of directional pleiotropy (*P* value for MR-Egger intercept > 0.05). MR-RAPS as a method for correcting for horizontal pleiotropy using robust adjusted profile scores in the IVW analyses was also reported. MR-RAPS can continuously estimate causal effects, even if some genetic variations violate the instrumental variable assumptions. Moreover, scatter plots depicting the relationship of the SNP effects on the exposure against the outcome were also provided. Therefore, ddifferent MR methods were employed where possible, and the consistent results could increase the reliability of MR estimates. Considering the problem of multiple tests, a 2-sided *P* value of < 0.01 (= 0.05/5 exposures) was deemed as statistically significant. All MR analyses were conducted based on the MR-base which is a database and analytical platform for MR being developed by the MRC Integrative Epidemiology Unit at the University of Bristol and/or Two Sample MR R package 24. The MR-RAPS package were used to conduct the MR-RAPS analyses which is the robust statistical inference for MR with many weak instruments 23.

### Pathway and functional enrichment analyses

Gene enrichment analysis is now well known a tool to explore the potential relationship between genes and phenotypes, although it does not reveal the exact mechanism, it can provide important reference information for subsequent mechanistic research. Functional annotation and pathway enrichment analyses, such as KEGG (https://www.kegg.jp/) and GO (http://geneontology.org/), were conducted with DAVID (https://david-d.ncifcrf.gov/) to preliminarily explore the mechanism of obesity affecting AF.

### Ethical approval

The present study made use of publicly available data, and did not require additional ethics approval.

## Results

### Association between adult obesity and AF

**Figure 2:**
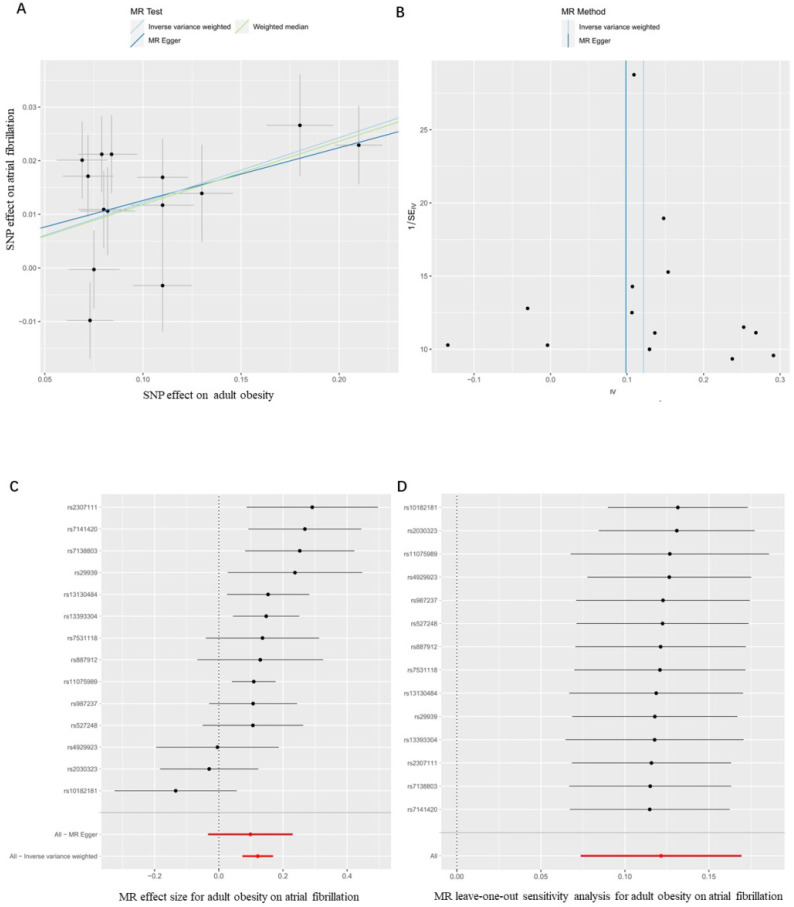
** MR results for the association of genetically adult obesity and atrial fibrillation.** (**A**) Method comparison; (**B**) funnel plot; (**C**) single SNP analysis; (**D**) leave-one-out analysis. SNP, single-nucleotide polymorphism; MR, Mendelian randomization.

Firstly, we evaluated the effect of adult obesity on AF, the IVW analysis suggested that the genetically predicted adult obesity was associated with the risk of AF, and 1-unit standard deviation (1-SD) increased in adult obesity was associated with a 13% higher risk of AF (*p* = 6.51×10^-7^, OR, 1.13 [95% CI, 1.08-1.19]) (Figure 2A). We used a funnel plot to assess the heterogeneity, larger spread suggests higher heterogeneity, which may be due to horizontal pleiotropy. Our funnel plot showed a small spread (Figure 2B). In addition, the visual inspection of the association of each SNP with adult obesity and its effect on AF was shown in Figure 2C, and the leave-one-out analysis suggested that the overall estimate was unlikely to be driven by any single SNP, as shown in Figure 2D. The sensitivity analyses, including the weighted median method, also supported this result (*p* = 1.24×10^-5^, OR, 1.13 [95% CI, 1.07-1.19]) which enhanced the reliability of the MR results, but the MR-Egger analysis inconsistent results with a wider CI (*p* = 1.67×10^-1^, OR, 1.10 [95% CI, 0.97-1.26]). However, the Egger regression intercept, which is an estimate of the magnitude of horizontal pleiotropy, was -0.08, and the directionality p-value was 0.12, indicating that no horizontal pleiotropy exists. MR-RAPS is a pleiotropy-corrected MR estimate method which corrected for horizontal pleiotropy in the IVW analyses by using robust adjusted profile scores. MR-RAPS as a more efficient and robust methods than many conventional methods, can continuously estimate causal effects, even if some genetic variations violate the instrumental variable assumptions. The result from MR-RAPS analyses also support this result (*p* = 1.88×10^-10^, OR, 1.13 [95% CI, 1.09-1.18]). To assess whether different types of adult obesity result in the same harm, we next evaluated the effect of adult WHRadjBMI on AF. The result from the MR-RAPS analysis and conventional MR analysis showed a possible causal relationship between adult WHRadjBMI and AF (*p* = 4.23×10^-3^, OR, 0.87 [95% CI, 0.79-0.96] for MR-RAPS analysis; *p* = 2.40×10^-2^, OR, 0.87 [95% CI, 0.78-0.98] for conventional MR analysis), but unfortunately, it didn't pass the multiple tests (*p* > 0.01) for conventional MR analysis (Supplementary Figure 1). Furthermore, results from the weighted median analysis and MR-Egger analysis also suggested no correlation between adult WHRadjBMI and AF (*p* > 0.01).

### Association between childhood obesity, childhood BMI and AF

Firstly, 5 SNPs that associated with childhood obesity in GWASs which included 13,848 participants were included in the MR analysis. The weighted median analysis and MR-RAPS analysis suggested a potential causal correlation between childhood obesity and AF (*p* = 6.89×10^-6^, OR, 1.12 [95% CI, 1.07-1.18] for weighted median analysis; *p* = 2.05×10^-6^, OR, 1.10 [95% CI, 1.05-1.14] for MR-RAPS analysis)**,** and the IVW analysis shown the similar result (*p* = 1.35×10^-2^, OR, 1.09 [95% CI, 1.02-1.17]), but it didn't pass the multiple tests (*p* > 0.01), MR-Egger analysis also showed inconsistent results (*p* > 0.01) (Supplementary Figure 2). Those divergent results among those methods may indicate that genetic pleiotropy is biasing some of these results.

**Figure 3:**
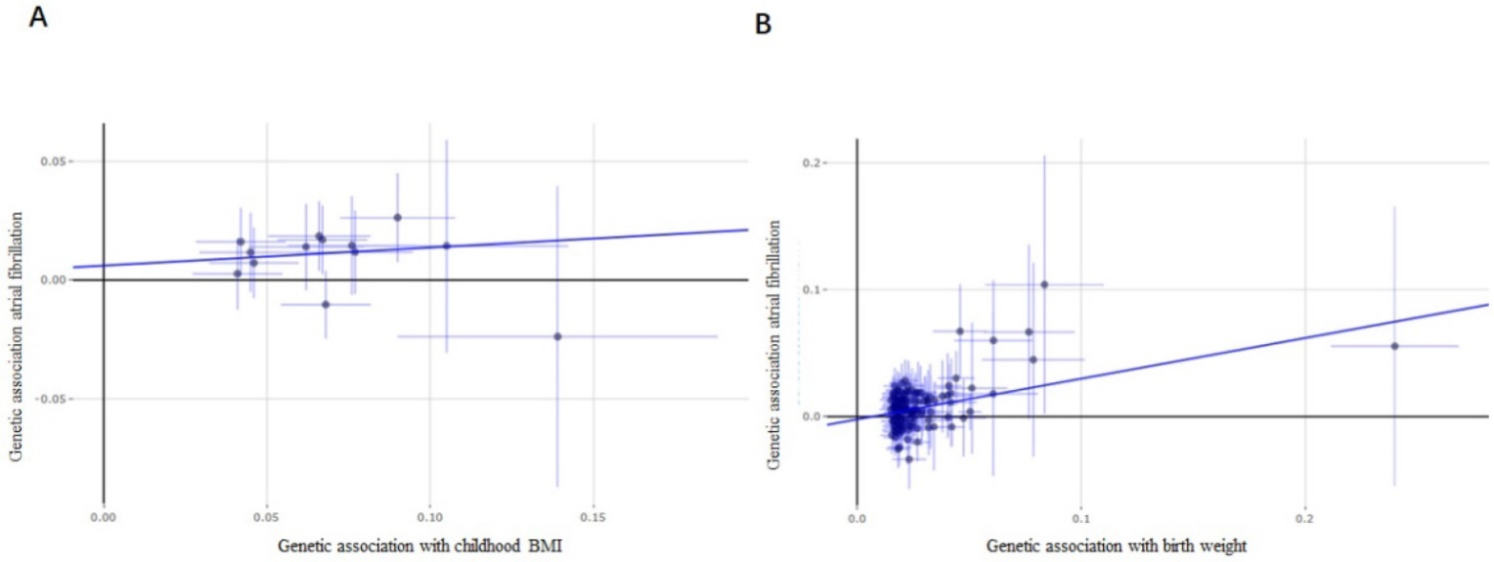
** Forest plot estimates the effect of genetically increased childhood BMI and birth weight risk on atrial fibrillation.** (**A**) Childhood BMI on atrial fibrillation; (**B**) birth weight on atrial fibrillation. BMI, body mass index.

Considering the limited number of SNPs and the limited sample sizes in this stage analysis which were insufficient to derive unbiased causal estimates. To maintain sufficient statistical power for our sensitivity analyses, we next retrieved a shortlist of SNPs associated with childhood BMI from a recent GWAS involving 47,541 people of European ancestry. Thirteen SNPs were included in the analysis, and similar results were found as those in the above analysis. Evidences from IVW analysis, weighted median analysis and MR-RAPS analysis suggested a potential causal association between childhood BMI and AF; 1-SD increase in childhood BMI was associated with an 18% higher risk of AF (*p* = 1.77×10^-4^, OR, 1.18 [95% CI, 1.08-1.29] for IVW analysis; *p* = 3.09×10^-5^, OR, 1.25 [95% CI, 1.13-1.39] for weighted median analysis; *p* = 8.90×10^-6^, OR, 1.19 [95% CI, 1.10-1.28] for MR-RAPS analysis) (Figure 3A), while MR-Egger analysis showed inconsistent results (*p* = 6.70×10^-1^, with a wider CI).

Both MR-Egger, weighted median and MR-RAPS analysis results for the causal association between childhood obesity, childhood BMI and AF were consistent, and the results from IVW were also similar (*p* = 1.35×10^-2^, OR, 1.09 [95% CI, 1.02-1.17] for childhood obesity;* p* = 1.77×10^-4^, OR, 1.18 [95% CI, 1.08-1.29]) for childhood BMI, but given the problem of multiple corrections, the results may be slightly different. However, considering that only 5 SNPs were included in the childhood obesity analysis, some deviations may have occurred, but they did not affect our conclusions.

### Association between birth weight and AF

**Figure 4:**
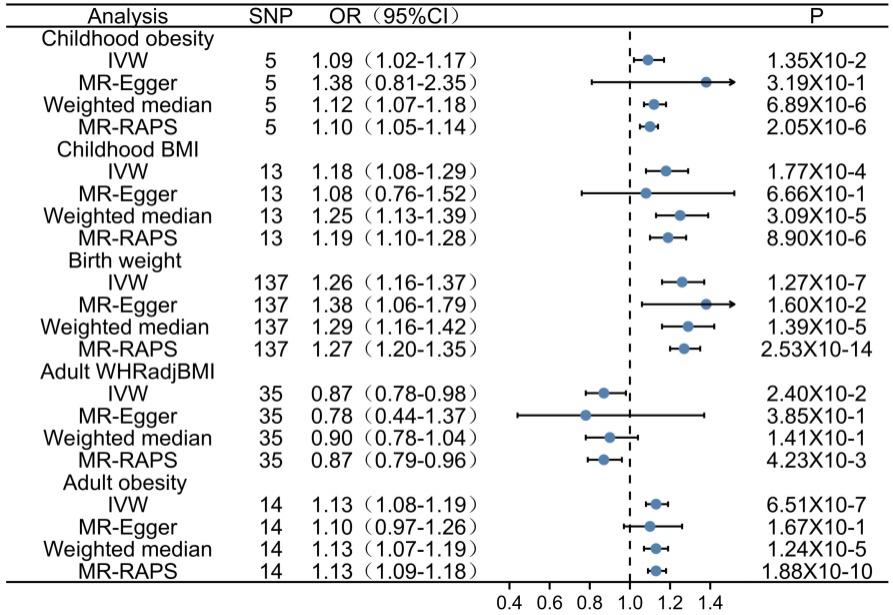
** Forest plot depicting MR results for the association of obesity-related traits with atrial fibrillation.** OR, odds ratio; CI, confidence interval; MR, Mendelian randomization; IVW, inverse variance-weighted; SNP, single-nucleotide polymorphism; BMI, body mass index.

By comparing the effects of 137 SNPs on birth weight and AF via conventional MR analysis, a causal relationship between birth weight and AF (*p* = 1.27×10^-7^, OR, 1.26 [95% CI, 1.16-1.37]) was observed, and a 1-SD increase in birth weight was related to a 26% increased risk of AF (Figure 3B). The association of birth weight with AF was consistent in complementary analyses performed using the weighted median analysis (*p* = 1.39×10^-5^, OR, 1.29 [95% CI, 1.16-1.42]), MR-Egger analysis (*p* = 1.60×10^-2^, OR, 1.38 [95% CI, 1.06-1.79]) (Figure 4), and MR-RAPS analysis (*p* = 2.53×10^-14^, OR, 1.27 [95% CI, 1.20-1.35]). There was no evidence of pleiotropy (MR-Egger intercept; β = -0.002, -0.008 to 0.004; *p* = 0.48), or heterogeneity between the MR estimates obtained from multiple SNPs (*I^2^ =* 78.90%). The results of these sensitivity analyses indicated that the pleiotropic effects might not affect our MR findings. A consistent effect was observed across various MR methods, which showed different assumptions regarding the causal effect of horizontal pleiotropy.

### Pathways and mechanisms

**Figure 5:**
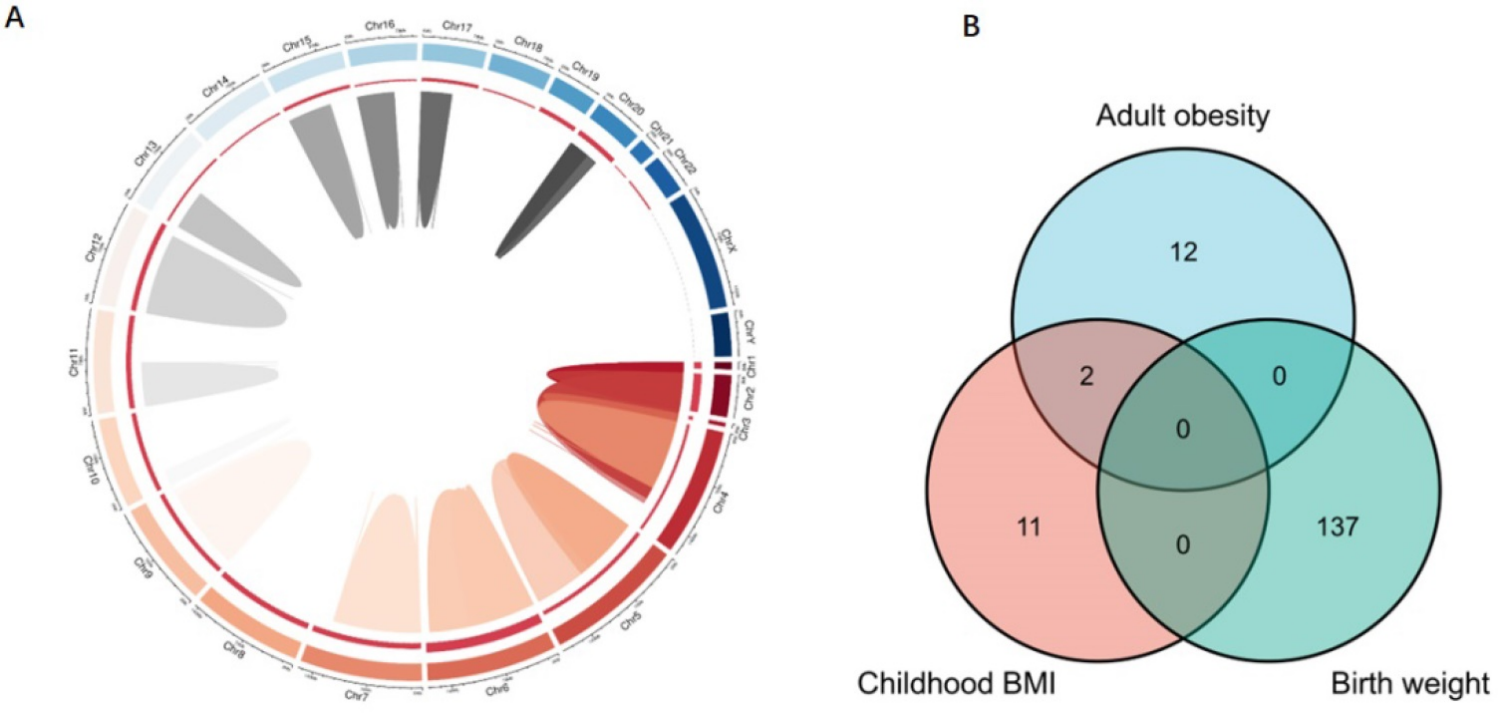
** Genes associated with obesity-related traits. (A)** The location of those genes in the genome; **(B)** the relationship between genes of different obesity-related traits. SNPs associated with children BMI, birth weight, and adult obesity and their associated genes distributed on multiple chromosomes. There are 2 SNPs overlaps between adult obesity and childhood BMI, while no overlaps were found in birth weight, adult obesity, and childhood BMI. SNP, single-nucleotide polymorphism; BMI, body mass index.

**Figure 6:**
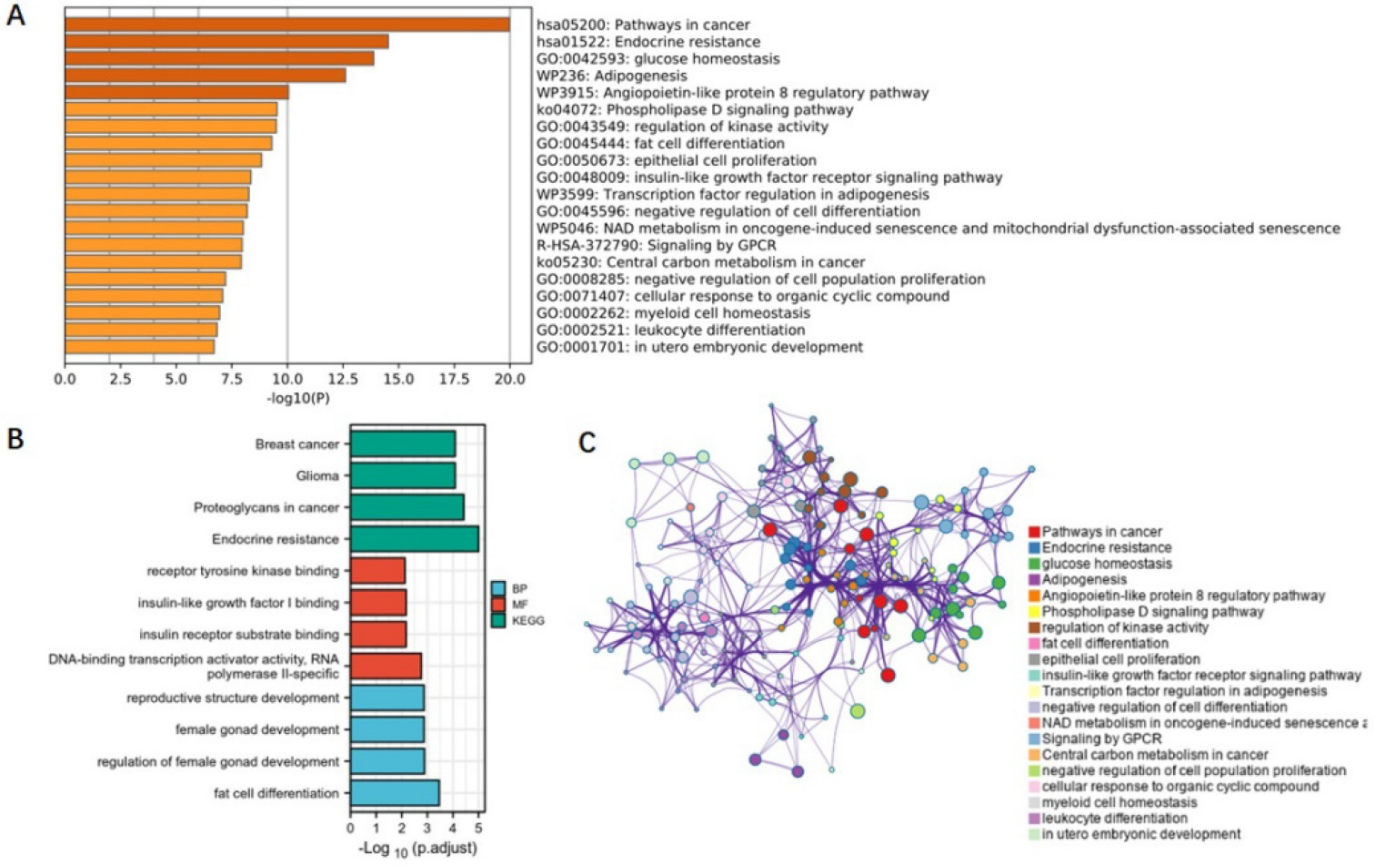
** Functional annotation and gene cluster analysis.** (**A**) Genes in the enrichment GO; (**B**) genes in the enrichment KEGG; (**C**) gene clusters and interactions between genes. SNPs associated with children BMI, birth weight, and adult obesity were functionally annotated. A total of 165 genes which are related to those SNPs were included in the gene cluster analysis. BMI, body mass index.

To clarify the mechanisms underlying the association between obesity-related traits and AF, we functionally annotated the SNPs associated with childhood BMI, birth weight, and adult obesity (Supplementary Table 1). The locations of SNPs and their associated genes in the genome are shown in Figure 5. There are 2 SNPs overlaps between adult obesity and childhood BMI, namely, rs987237 (*TFAP2B*) and rs13130484 (*GNPDA2*), but no overlaps were found in birth weight, adult obesity and childhood BMI, which means that some childhood obesity may continue into adulthood, while birth weight has a limited effect on childhood or adult obesity. *GNPDA2* has been reported to be associated with influencing BMI and susceptibility to obesity 25. Studies have also suggested that *TFAP2B* is associated with BMI in both Europeans and African Americans 26, 27. Birth weight, childhood obesity and adult obesity may have common causes as well as different ones, but considering that they all increase the risk of AF, we included all relevant SNPs of these three traits in the analysis to determine their possible mechanism for causing AF. A total of 165 genes which are related to those SNPs were included in the gene cluster analysis (DAVID). GO and KEGG databases were employed to determine the molecular pathways associated with these genes. GO (Figure 6A) and KEGG (Figure 6B) enrichment both demonstrated that the genes were mainly enriched by insulin-like growth factor receptor signaling, endocrine resistance, glucose homeostasis, adipogenesis, fat cell differentiation, and pathways related to cancer. In addition, gene clusters and interactions between genes shown in the Figure 6C indicated the same results. The results of our gene cluster analysis through multiple enrichment methods indicate the important role of metabolism in AF. Although gene enrichment analysis is now well known as a tool to explore the potential relationship between genes and phenotypes, further experiments may be needed to confirm the exact mechanism and distinguish the different mechanisms between different obesity-related traits.

## Discussion

In this study, two-sample univariable MR was used to estimate the effect of obesity-related traits at different stages (birth weight, childhood BMI, adult obesity) on AF. The results indicated that 1-SD increases in adult obesity, childhood BMI and birth weight were associated with 13%, 18% and 26% higher risks of AF (Graphical Abstract), respectively, suggesting that earlier age at obesity is associated with higher AF risk.

Obesity is an independent risk factor for AF 28. Clinical and epidemiological research has suggested that obesity induces the development, progression and recurrence of AF 29. Berkovitch et al. demonstrated that every 1 kg/m^2^ increase in BMI was associated with a 7% higher risk of AF within a large Israeli cohort of 18,290 participants. In line with previous clinical and epidemiological observational research, an MR study indicated that higher BMI in adult causally increased the risk of AF 30. This would be consistent with our results that 1-SD increases in adult obesity were associated with a 13% higher risk of AF. Although a high BMI is consistently associated with AF risk, the opposite and counter-intuitive results have been observed for mortality. For example, mildly obese (BMI: 30-35kg/m^2^) and overweight (BMI: 25-30kg/m^2^) individuals tended to have lower risk of all-cause mortality during long-term follow-up 31, 32. There are numerous potential causes that may explain this obesity paradox, and BMI alone may not accurately predict the risk of AF. Hence, the type of obesity should also be considered. In this study, we not only determined the effect of BMI on AF, but also evaluated the impact of adult WHRadjBMI on AF. We found no causal relationship between adult WHRadjBMI and AF, and the results showed a negative correlation trend. This finding suggests that different types of obesity may have different effects on AF, and obesity types should be distinguished in the risk prediction of AF.

It is worth to notice that the incidence of AF in younger patients has increased due to unhealthy lifestyles, premature hypertension, obesity and cardiac disease 33. A population-based cohort study of Danish school children showed that among the 314,140 children aged 7-13 years, 17,594 were diagnosed with AF during adulthood 34. Consistent with this finding, our study showed that 1-SD increases in childhood BMI were associated with the 18% higher risks of AF, and a previous MR study demonstrated that a consistent association between genetically predicted childhood obesity and an increased risk of AF in adult 35.

Current studies on the association between birth weight and AF are conflicting. In a cohort of female health professionals, a high birth weight was associated with an increased risk of AF, but the association was attenuated after adjustment for adult height 36. In contrast, the Atherosclerosis Risk in Communities cohort found a higher risk of AF in individuals with a low birth weight 37. It has even been found that both high birth weight and low birth weight (in men) were associated with an increased risk of AF 38. Recently, Chen et al. suggested that elevated birth weight was associated with an increased lifelong risk of AF, which may be partially mediated by BMI 7. The reason for these inconsistent outcomes is unknown and requires further investigation.

Our study suggested that obesity at different stages has different effects on AF, and the higher the early-life BMI, the greater the impact on AF. The earlier obesity onset is, the less it is affected by environmental factors, and the more accurate it is to evaluate AF. However, adult obesity and childhood obesity in our MR analysis were considered as coarsened exposures, which will result in inflated or deflated effect estimates. Recently, Tudball et al. proposed a framework to try to clarify this violation, and their result showed that coarsened exposures may inflate or deflate effect estimates but will not reverse their direction 39. Although our results provide evidence of a potential causal relationship between adult and childhood obesity and AF, their effect values may be underestimated or overestimated. It is also unclear how much the SNPs included in our analysis explain the variance of adult and childhood obesity respectively. This makes it more difficult to accurately assess the effect of obesity on AF. A growing body of evidence suggests that early obesity or elevated BMI may increase the risk of AF, although the actual effect may not have been accurately assessed. In addition, separating the independent effects of childhood and adulthood obesity on AF risk is challenging as children with obesity typically remain overweight throughout the life course. Power, G.M., et al. identified strong evidence of a total effect between genetically predicted childhood body size and increased risk of AF by univariable analyses. However, evidence of a direct effect was weak after accounting for adult body size using multivariable MR, suggesting that childhood body size indirectly increases risk of AF outcomes via the pathway involving adult body size 35. Further research is necessary to ascertain the critical timepoints where if ever, the detrimental impact of obesity initiated in early life begins to become immutable.

The relationship between obesity and AF are complex. Obesity shares common pathophysiological pathways with AF. Although BMI is a strong predictor of AF 40, much recent interest has focused on epicardial fat. Epidemiological and clinical studies have demonstrated that epicardial fat is consistently associated with the presence, severity, and recurrence of AF 41-43. Three potential mechanisms could be involved in the processes: 1) Adipocyte of epicardial fat infiltration into the atrial myocardium could directly result in conduction slowing or anisotropy akin to that seen with microfibrosis 44, 45. 2) The accumulation of adipokines secreted from epicardial fat within the pericardial sac may facilitate paracrine effects on the atrial myocardium that promote fibrosis 46. 3) Markers of inflammation (e.g., C-reactive protein, IL-6, IL-8, IL-1b, and TNF-a) are secreted by epicardial fat, and may have local pro-inflammatory effects on the adjacent atrial myocardium that facilitate arrhythmogenesis 47.

Interestingly, our study demonstrated that earlier age at obesity onset was associated with an increased risk of AF. We found that there were different genetic factors that could lead to obesity at different stages. Thus, it can be inferred that various mechanisms are involved in obesity during different stages. Notably, there were two SNPs overlapping between adult obesity and childhood BMI, namely, rs987237 (*TFAP2B*) and rs13130484 (*GNPDA2*), but no overlaps were found in birth weight, adult obesity and childhood BMI, which means that some childhood obesity may continue into adulthood, while birth weight has a limited effect on childhood or adult obesity. Further experiments may be needed to confirm the exact mechanism and distinguish the different mechanisms between different obesity-related traits. Previous studies have suggested that TFAP2B is associated with BMI in both Europeans and African Americans 26, 27. Moreover, genetic variants within *TFAP2B* have recently been reported to correlate positively with TFAP2B transcript levels in adipose tissue 48 and TFAP2B may be involved in global adipocyte response to positive energy balance 49. This study highlights the shared genetic background between childhood and adult BMI. TFAP2B likely represents age-related differences in strength of the associations with BMI 19. *GNPDA2* has also been reported to be associated with influencing BMI and susceptibility to obesity. However, the association between *TFAP2B*, *GNPDA2*, and AF has been less well studied. In this context, it may be speculated that *TFAP2B* and *GNPDA2* as the mediators of the BMI-associated increase the risk of AF. Further research is necessary to ascertain the critical timepoints where, if ever, the detrimental impact of obesity initiated in early life begins to become immutable.

In this study, GO and KEGG analyses suggested that most of the enriched genes were not only primarily involved in metabolic processes but also involved in cancer progression. An association between AF and malignancy has been reported 50. AF risk is increased after treatment with chemotherapy or surgery. More importantly, AF appears more frequently in cancer patients at the time of diagnosis and before receiving therapy 51, 52, suggesting an overlap in pathophysiological processes 53. The multifactorial mechanisms underlying the interrelationship between obesity and AF risk include shared risk factors, a higher risk of bleeding with anticoagulants, or other systemic processes.

The advantages of this study were (i) the use of data from large-scale GWASs with sufficient statistical power and (ii) the study design of two-sample MR analysis for determining the association between obesity-related traits and AF risk. More importantly, most subjects of European descent were included in the GWASs selected for MR analyses, which could reduce potential bias attributable to population stratification. Additionally, this method was less likely to be affected by unmeasured confounders and reverse causation, as the genetic materials might have no negative effects on a common exposure. Potential limitations of this study were that (i) the SNPs could be associated with confounding factors and (ii) a litter sample for obesity-related traits and AF overlapped with each other in GWASs, which could introduce bias in the MR estimations of obesity-related traits and AF, thus, affecting the reliability and accuracy of MR results. However, only litter samples for birth weight and AF GWAS are overlapped, which both from the UK Biobank, the overlapping samples accounted for less than 5% of the samples, and given factors such as genotyping and age, the actual overlap may be even less. In addition, our findings were based on strict MR assumptions, and were robust across different MR methods. Therefore, it does not affect our conclusions. Additionally, since there was a small number of SNPs for some of our exposures, it might make more sense to consider weak instrument MR techniques such as MR-GRAPPLE analysis which can efficiently use both strong and weak genetic instruments, detect the existence of multiple pleiotropic pathways, determine the causal direction and perform multivariable MR to adjust for confounding risk factors 54, 55. Although the results of various methods all support our conclusion, the support of MR-GRAPPLE results will make our conclusion more convincing. However, due to the inadequacy of some of the raw GWAS summary data for exposures and the limitations of our conditions, we were not able to conduct the MR-GRAPPLE analysis.

## Conclusions

Our study indicated that different stages of obesity were associated with AF risk. An earlier age at obesity onset contributed to a higher risk of AF. There was no overlap in the greater risk of early obesity for AF, suggesting that clinicians should pay more attention to obesity in children or infants than they do in adults. Nevertheless, further studies with larger sample sizes are warranted to elucidate the exact mechanisms underlying the association of childhood and adult obesity with AF risk.

## Supplementary Material

Supplementary figures.

## Figures and Tables

**Table 1:** Description of GWAS consortiums for phenotypes

Phenotype	First author (year)	Consortium	Sample size	Population, age
Childhood obesity	Bradfield (2012)	EGG	Cases= 5,530 Controls= 8,318	All European, 2 to 18 years
Childhood BMI	Felix (2016)	EGG	47,541	All European, 2 to 10 years
Birth weight	Warrington (2019)	EGG and UK Biobank	321,223	92.81% European, birth
Adult obesity	Berndt (2013)	GIANT	263,407	All European, >19 years
Adult WHRadj BMI	Shungin (2015)	GIANT	224,459	All European, >19 years
Atrial fibrillation	Roselli (2018)	AFGen and Broad AF, UK Biobank and Biobank Japan	Cases= 65,446; Controls=522,744	91.37% European, >19 years
