# Supplementary material for: The Risk of Atrial Fibrillation Increases with Earlier Onset of Obesity: A Mendelian Randomization Study
Source: Int J Med Sci. 2022 Aug 8;19(9):1388–98. doi: 10.7150/ijms.72334 (PMC9413561; doi:10.7150/ijms.72334)
Supplement: Supplementary file 1 — Supplementary figures. [file ijmsv19p1388s1.pdf]

Supplementary Material

Supplementary Figures and Tables

Table S1 SNPs included in MR studies

SNP	Chr	Pos	Gene names
**SNPs related to adult obesity**			
rs8028313	15	68043057	*MAP2K5*
rs887912	2	59302877	*FANCL*
rs9816226	3	185834499	*DGKG*
rs987237	6	50803050	*TFAP2B*
rs10182181	2	25150296	*ADCY3*
rs11075989	16	53819877	*FTO*
rs13130484	4	45175691	*GNPDA2*
rs13393304	2	637830	*TMEM18*
rs2030323	11	27728539	*BDNF*
rs2307111	5	75003678	*POC5*
rs29939	19	34310800	*KCTD15*
rs523288	18	57848369	*U4*
rs527248	1	177875514	*SEC16B*
rs7138803	12	50247468	*FAIM*
rs7141420	14	79899454	*NRXN3*
rs7531118	1	72837239	*NEGR1*
rs4929923	11	8639200	*TRIM66*
**SNPs related to childhood BMI**			
rs13130484	4	44870448	*GNPDA2*
rs11676272	2	24995042	*ADCY3*
rs4854349	2	637861	*TMEM18*
rs543874	1	176156103	*SEC16B*
rs7132908	12	48549415	*FAIM2*
rs12429545	13	53000207	*OLFM4*
rs987237	6	50911009	*TFAP2B*
rs12041852	1	74776088	*TNNI3K*
rs13253111	8	28117893	*ELP3*
rs8092503	18	50630485	*RAB27B*
rs3829849	9	128430621	*LMX1B*
rs13387838	2	206989692	*ADAM23*
rs7550711	1	109884409	*GPR61*
**SNPs related to birth weight**			
rs1482852	3	156798294	*LOC339894/CCNL1*
rs138715366	7	44246271	*YKT6/GCK*
rs7968682	12	66371880	*HMGA2*
rs17034876	2	46484310	*EPAS1*
rs35261542	6	20675792	*CDKAL1*
rs11708067	3	123065778	*ADCY5*

rs2174633	4	17917781	*LCORL/DCAF16*
rs7772579	6	152042502	*ESR1*
rs28457693	9	98217348	*PTCH1/FANCC*
rs1801253	10	115805056	*ADRB1*
rs11698914	20	31327144	*COMMD7*
rs2131354	4	145599908	*LOC646576/HHIP*
rs1112718	10	94479107	*HHEX/IDE*
rs2428362†	17	7180274	*CLDN7/SLC2A4*
rs11042596	11	2118860	*INS-IGF2*
rs112139215	7	73034559	*MLXIPL*
rs1012167	20	39159119	*MAFB*
rs1480470	12	66412130	*HMGA2*
rs3184504	12	111884608	*SH2B3*
rs7402983	15	99193276	*IGF1R*
rs9645500	10	70986723	*HKDC1/HK1*
rs13266210	8	41533514	*ANK1*
rs2274224	10	96039597	*PLCE1*
rs55958435	15	96852638	*NR2F2*
rs2551347	2	23912401	*KLHL29*
rs10985827	9	125701608	*RABGAP1/GPR21*
rs6533183	4	106133184	*TET2*
rs854037	5	57091783	*ACTBL2*
rs11893688	2	9695282	*ADAM17*
rs28505901	9	139241030	*GPSM1*
rs4444073	11	10331664	*ADM*
rs71486610	10	124134803	*PLEKHA1*
rs34776209	7	23513093	*IGF2BP3*
rs2418135	9	113901309	*LPAR1*
rs3933326	9	123633948	*PHF19*
rs9379832	6	26186200	*HIST1H2BE/HIST1H2BH*
rs72656010	8	57122215	*PLAG1*
rs75034466	6	34199815	*HMGA1*
rs2934844	6	166142456	*PDE10A*
rs7223535	17	29211667	*ATAD5*
rs9348981	6	35687249	*FKBP5/MAPK13/TEAD3*
rs116807401	4	135121721	*PABPC4L*
rs10935733	3	148622968	*CPA3/AGTR1*
rs4719648	7	2756832	*AMZ1/GNA12*
rs4932373	15	91429287	*FES/FURIN*
rs41311445	22	42070374	*NHP2L1/SREBF2*
rs6930558	6	141878920	*NMBR*
rs2306547	12	26877885	*ITPR2*
rs6040076	20	10658882	*JAG1*
rs7819593	8	106115172	*ZFPM2*
rs10283100	8	120596023	*ENPP2*
rs9549046	13	40647206	*LINC00332*

rs6575803	14	101257755	*MIR2392/DLK1*
rs10181515	2	227019461	*LOC646736/COL4A4/IRS1*
rs8106042	19	7161849	*INSR*
rs9851257	3	123125711	*ADCY5*
rs2647873	12	103081192	*LINC00485/IGF1*
rs11055030	12	12878349	*APOLD1*
rs9318511	13	78601413	*LINC00446*
rs1547669	6	33775641	*MLN*
rs80278614	1	119412317	*TBX15*
rs62496903	8	6446938	*MCPH1*
rs670523	1	155878732	*RIT1/LMNA*
rs667515	11	69449076	*CCND1*
rs516246	19	49206172	*FUT2*
rs73354194	17	79905947	*MYADML2*
rs7854962	9	96900505	*PTPDC1*
rs2946179	5	157886627	*EBF1*
rs732563	8	23345526	*ENTPD4/NKX3-1*
rs255773	19	54723546	*LILRB3/RPS9*
rs6467157	7	127660763	*SND1*
rs6911621	6	35529025	*FKBP5/MAPK13/TEAD3*
rs7744700	6	53349401	*GCLC*
rs28365970	5	67585723	*PIK3R1*
rs2282978	7	92264410	*CDK6*
rs73143584	20	62445702	*ZBTB46*
rs78378222	17	7571752	*TP53*
rs13257363	8	142252580	*SLC45A4*
rs9909342	17	25652275	*WSB1*
rs13271368	8	126506140	*TRIB1*
rs3806315	1	214724668	*PTPN14*
rs10830963*	11	92708710	*MTNR1B*
rs9366778	6	31269173	*HLA-C*
rs12401656	1	43456767	*FLJ32224/SLC2A1*
rs4953353	2	46567276	*EPAS1*
rs72480273	1	161644871	*FCGR2B/FCGR2C/HSPA6*
rs220193	21	43581308	*UMODL1*
rs75844534	15	38667117	*SPRED1*
rs1323438	9	119115531	*PAPPA*
rs34217484	13	48854550	*LINC00441/RB1*
rs2229742	21	16339172	*NRIP1*
rs2779165	19	4915447	*UHRF1*
rs1981627	5	133838180	*PHF15*
rs34036147	8	38366249	*C8orf86/FGFR1*
rs11867479	17	68090207	*KCNJ16*
rs41355649	19	33790556	*CEBPA*
rs10883846	10	104958244	*NT5C2/CYP17A1*
rs10913200	1	176521655	*PAPPA2*
rs339969	15	60883281	*RORA*

rs6026449	20	57272617	*STX16-NPEPL1/GNAS*
rs40434	16	55699525	*SLC6A2*
rs11711420	3	183349010	*KLHL24*
rs2168443	3	46947087	*PTH1R*
rs3740360	10	96025491	*PLCE1*
rs11082304	18	20720973	*CABLES1*
rs61885091	11	69791952	*ANO1/FGF4*
rs6033062	20	11207419	*LOC339593*
rs134594	22	29468456	*KREMEN1*
rs72681869	14	50655357	*SOS2*
rs6569647	6	130337266	*L3MBTL3*
rs754868	2	43185532	*HAAO*
rs2280235	2	191843830	*STAT1*
rs1203876	20	22540915	*LINC00261/FOXA2*
rs2889874	20	33715777	*EDEM2/MYH7B*
rs6582623	12	46613394	*SLC38A1*
rs61830764	1	212289976	*DTL*
rs10265057	7	47275737	*TNS3*
rs1415701	6	130345835	*L3MBTL3*
rs234864	11	2857297	*KCNQ1*
rs2306700	3	142123841	*XRN1*
rs59084784	7	22739562	*IL6*
rs708122	1	228216997	*WNT3A*
rs1129156	19	40719076	*MAP3K10/AKT2*
rs7285579	22	46441980	*LOC100271722*
rs186606513	2	97482001	*CNNM3*
rs5030317	11	32410337	*WT1*
rs147957154	19	43431040	*PSG7*
rs351930	5	52003397	*PELO*
rs11983722	7	46298647	*IGFBP3*
rs9267812	6	32128394	*PPT2*
rs753381	20	39797465	*PLCG1*
rs4350272	10	25056118	*ARHGAP21*
rs962554	6	142734204	*GPR126*
rs1818782	5	39424628	*DAB2*
rs2237467	7	50733316	*GRB10*
rs2045457	16	20046115	*GPR139/GPRC5B*
rs6925689	6	126865884	*CENPW*

Supplementary Figures

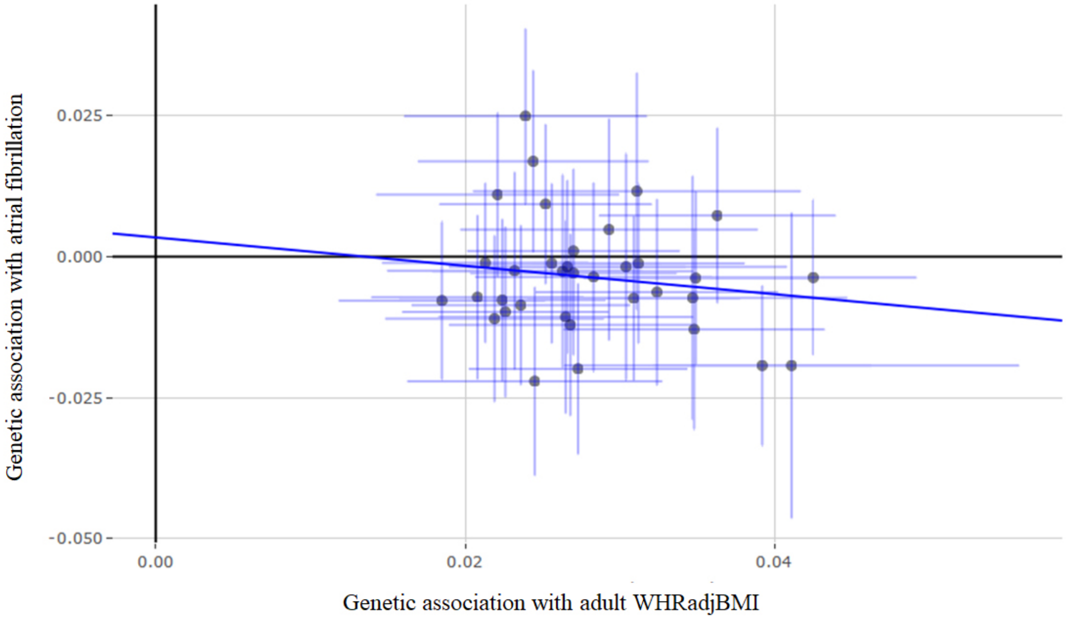

Figure S1. The effect of adult WHRadjBMI on atrial fibrillation. Forest plot estimates the effect of genetically increased adult WHRadjBMI risk on atrial fibrillation.

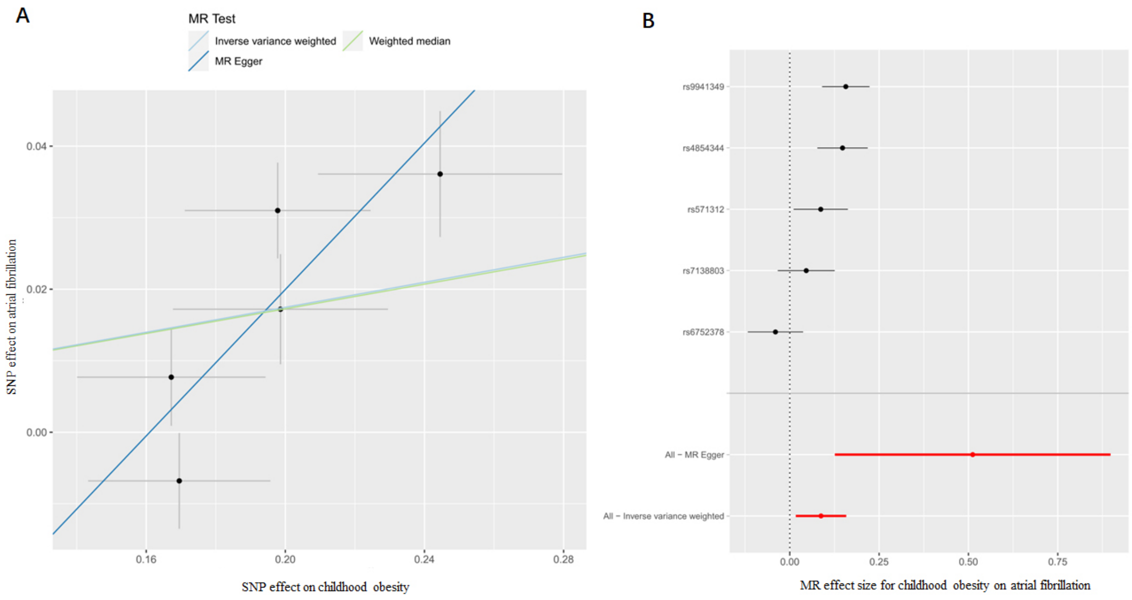

Figure S2. The effect of childhood obesity on atrial fibrillation.

MR results for the association of genetically childhood obesity and atrial fibrillation. (A) method comparison; (B) single SNP analysis.
